# The complete genome of “*Candidatus* Phytoplasma fraxini” AshY1 from the ash yellows group

**DOI:** 10.1128/mra.00318-24

**Published:** 2024-06-11

**Authors:** Jan W. Böhm, Bruno Huettel, Bernd Schneider, Michael Kube

**Affiliations:** 1 Integrative Infection Biology Crops-Livestock, University of Hohenheim, Stuttgart, Germany; 2 Max Planck Genome-centre, Max Planck Institute for Plant Breeding, Köln, Germany; 3 Institute for Plant Protection in Fruit Crops and Viticulture, Julius Kühn-Institut, Federal Research Centre for Cultivated Plants, Dossenheim, Germany; The University of Arizona, Tucson, Arizona, USA

**Keywords:** 16SrVII, *Fraxinus*, genomics

## Abstract

The complete genome of “*Candidatus* Phytoplasma fraxini” AshY1, originating from *Fraxinus americana* in North America, was assembled using long reads from single-molecule real-time sequencing technology. The chromosome of 598 kb provides insights into the effector repertoire of a phytopathogenic bacterium from the 16SrVII phytoplasma group.

## ANNOUNCEMENT

The obligate biotrophic bacteria of “*Candidatus* Phytoplasma” are limited to the plant phloem and insects. Many of the phytopathogenic members can cause chlorosis in plants, including the ash-yellow pathogens of the 16SrVII group. “*Candidatus* Phytoplasma fraxini” was observed in several ash species (*Fraxinus* spp.) in North and South America. Associated symptoms are yellowing, shoot proliferation, and suppressed root growth on ash and lilac species (*Syringa* spp.) (1). Besides detections in ash, other tree species of the family *Euphorbiaceae*, *Magnoliaceae*, *Salicaceae,* and *Fagaceae* have been described as hosts (2). However, the host range is not restricted to woody plants, as “*Candidatus* Phytoplasma fraxini” has also been detected in potatoes (2–4).

The AshY1 strain of “*Candidatus* Phytoplasma fraxini” originated in Ithaca (New York, United States) in white ash (*Fraxinus americana*) and was transferred to *Catharanthus roseus* (5). The sequencing template was metagenomic DNA prepared from infected *C. roseus* shoot and leaf material using the DNeasy Blood & Tissue Kit (Qiagen, Hilden, Germany). A high-fidelity library for single-molecule real-time (SMRT)-sequencing was prepared using SMRTbell prep kit 3.0 (Pacbio, California, USA) without additional DNA fragmentation. The fragment library was sequenced on a Sequel IIe device (Pacbio) at the Max Planck Genome-Centre (Cologne, Germany) with Binding Kit 2.0 (Pacbio) and Sequel II Sequencing Kit 2.0 (Pacbio). A total of 310,838 metagenomic reads were obtained, of which 11,518 reads (N_50_ of 5834) were assigned to the genus “*Candidatus* Phytoplasma” by taxonomic binning using BLAST+ v2.2.9, Metagenome Analyzer (MEGAN) v.6.18.2 (6), and a custom database of assigned nucleotide data entries from “*Candidatus* Phytoplasma” and *Catharanthus roseus* in GenBank (accession: January 2024). The remaining reads were assembled with Canu v2.2 (7) using the pacbio-hifi option and an estimated genome size of 600 kb. A contiguous, circular sequence with a 67.17-fold coverage was achieved. Sequence overlap of >10 kb was confirmed through BLAST analysis. Subsequently, the sequence overlap was manually removed using Artemis v18.2.0 (8). Annotation of the complete chromosome was performed in RAST v2.0 (9) followed by manual curation in Artemis v18.2.0 (8), with *dnaA* set as the first gene of the chromosome. The completeness of the annotation is supported by the comparison with 151 single-copy orthologs (94%) from the Mollicutes group using BUSCO (10). Reads not considered in the chromosome assembly were subjected to an additional taxonomic binning and screened for extrachromosomal DNA of AshY1. No plasmid was identified. Default parameters were used for all software unless otherwise specified.

The genome of “*Candidatus* Phytoplasma fraxini” AshY1 consists of one circular chromosome with 598,519 bp in length, a GC content of 23.24%, 32 tRNAs, 512 protein-coding genes, and 23 pseudogenes. Interestingly, the genomic content of AshY1 encodes insect vector-associated membrane proteins (e.g., VmpB) and a putative shoot proliferation-inducing effector (e.g., SAP11-like), which are shared with closely related 16SrV phytoplasmas (11).

## Data Availability

Raw SMRT-reads have been deposited in Sequence Read Archive under accession SRR28122442. A whole-genome project has been generated in GenBank under BioProject accession number PRJNA1081420 and annotation is available under accession CP146843.
